# Feasibility and thematic analysis of narrative visualization materials with physical activity monitoring among breast cancer survivors

**DOI:** 10.1186/s12885-022-09629-7

**Published:** 2022-05-16

**Authors:** Jason R. Bentley, Xiaoying Yu, Amol M. Karmarkar, Brian Downer, John Prochaska, Elizabeth J. Lyons

**Affiliations:** 1 University of Texas Medical Branch, Galveston, TX USA; 2 University of Houston Clear Lake, Houston, TX USA; 3 Virginia Commonwealth University, Richmond, VA USA

**Keywords:** Physical activity behavior, Narrative visualization, Scrapbooking, Breast cancer survivors

## Abstract

**Background:**

Breast cancer survivors have a unique risk for negative health outcomes. Engaging in routine physical activity (PA) can reduce these risks. However, PA levels are low among this population. Narrative visualization (NV) is a technique that uses drawings, photographs, and text to contextualize data, which may increase integrated regulation, or motivation related to personal values and identity. A PA intervention targeting breast cancer survivors using an NV strategy may improve PA behavior. The purpose of this study was to determine whether scrapbooking activities could successfully be used as an NV strategy for older (55+) breast cancer survivors.

**Methods:**

Breast cancer survivors were given workbooks, wearable electronic activity monitors, instant cameras, and art supplies including a variety of stickers (e.g., emojis, affirmations). Participants were instructed to use these materials for 7 days. The workbook pages prompted participants to re-draw their daily activity graphs from the wearable’s mobile app, then annotate them with text, photographs, stickers, etc. to reflect what the data meant to them. Hybrid thematic analysis was used to analyze the photographs, drawings, and written content to identify emergent themes. Content analysis was also used to investigate use of stickers and photographs.

**Results:**

Of the 20 consented women (mean age 67 ± 5 years, 45% non-Hispanic white), 3 participants were lost to follow-up or unable to complete the procedures. The NV procedures were successfully utilized by the remaining 17 participants, who collectively used 945 stickers over 7 days, most of which were emojis. Emojis were both positively and negatively valanced. Participants took a mean of 9 photos over 7 days and completed workbook questions regarding current PA and PA goals. Themes within the photos included family, specific locations, everyday objects, religion, and friends. Themes within the written portions of the workbook included family, chores and obligations, health, personal reflection, hobbies, and shopping.

**Conclusions:**

The materials provided allowed breast cancer survivors to successfully use NV techniques to reflect on their PA data and behavior. These techniques show promise for promoting integrated regulation in activity monitoring interventions.

**Trial registration:**

This study was funded by the National Cancer Institute (R21CA218543) beginning July 1, 2018.

## Background

To reduce the risk of disease recurrence, protect against long-term treatment side effects as well as deficits in health-related quality of life, it is recommended that breast cancer survivors engage in at least 150 minutes per week according to the American Cancer Society (ACS) [1]. Moreover, considering that the majority of breast cancer survivors are over age 65, physical activity may also reduce or manage age-related comorbidities such as cardiovascular disease [2, 3]. In fact, based on a large body of observational studies, physical activity is associated with reduced all-cause and breast cancer-specific mortality [4]. An equally impressive evidence base of randomized controlled trials points to the positive effects of physical activity on physiology as well as subjective measures of psychological outcomes and quality of life [5]. While the evidence for the beneficial associations and effects of exercise and physical activity is overwhelming [6, 7], the literature also highlights that adherence to the ACS physical activity guidelines is problematic, between 16 and 35% [8, 9]. For instance, while estimates vary based on specific study condition, it is generally believed that only 35% of survivors meet the ACS recommendations and that adherence is especially low among older breast cancer survivors [9, 10]. Of note, adherence to the standard 150 minutes/week of moderate-level physical activity (PA) is lower in breast cancer patients than US adults (49%) including those 65 and older (39%) [11]. Similarly, adherence, particularly long-term adherence (1 year or longer), to even supervised PA interventions and exercise programs is low among breast cancer survivors, especially older breast cancer survivors [9].

Because barriers to adherence are widely identified, interventions have been designed to address participants’ motivational factors to increase habitual PA and thus, improve the long-term health of breast cancer survivors, particularly those who are at higher risk for non-adherence (e.g., older and more obese survivors). A common strategy in the motivation literature draws on psychological processes related to self-regulation and self-efficacy which may play an important role in PA engagement for breast cancer survivors including adherence [12]. Self-regulation is the process of guiding one’s own behaviors to reach goals, particularly by managing disruptive emotions or impulses [13]. Essentially, this is goal-driven behavior where the long-term goal drives behavioral choices even when feeling fatigued or otherwise tempted to give up. Similarly, self-efficacy relates to one’s beliefs in their own capabilities to successfully engage in a predetermined course of action [14]. PA interventions based on self-regulation typically involve setting PA goals, monitoring PA, and receiving meaningful feedback on progress [15]. With the increasing usage of wearable electronic activity monitors (e.g. FitBit), this feedback most often takes the form of displays showing percentages toward a predetermined goal. The software interface for these devices also typically shows charts of steps per day, comparisons to previous days, as well as weekly and monthly trends [16].

Interventions that use goal setting, self-monitoring, and routine feedback appear to produce short-term increases in PA, but rarely do they accomplish long-term behavioral change [17–19]. These programs may be limited by lack of guidance in the use and interpretation of the self-monitoring charts and data. Several studies have suggested that a lack of such guidance is a major reason why individuals stop using PA monitors [20, 21]. Breast cancer survivors have also reported that they reduced adherence to self-regulation behaviors once they were no longer accountable to interventionists [22]. This population further reports that they want their PA goals to be contextualized as part of their larger, value-based life goals [23, 24]. Breast cancer survivors also face unique barriers related to their identities. Changes to the body, particularly body parts associated with femininity, can greatly alter perceptions of identity [18, 25], which may in turn impact PA.

Self-Determination Theory (SDT) provides a framework for a more motivation-related perspective on behavior change intervention [19]. This theory postulates that motivation can range from fully internal or intrinsic, which is one’s inherent drive to seek out challenges, to fully external or extrinsic, where motivation comes entirely from external sources. Higher levels of more autonomous forms of motivation (e.g., closer to intrinsic) tend to predict long-term, self-regulated adherence to PA [26]. Thus, it is critical to establish autonomy when PA is being introduced and integrated into routine behavior [27]. The most autonomous type of extrinsic regulation is called integrated regulation, or motivation related to personal values and identity. Integrated regulation is associated with engagement in regular PA over time [28]. While this form of targeted motivation is understudied in older adult cancer survivors, several studies suggest that it may address limitations in current intervention strategies for PA maintenance in this population [29–31]. However, while standard self-regulation interventions are well-suited to produce behavior change initiation, they are not capable of facilitating long-term PA maintenance [32].

When it comes to self-monitoring PA, using less numerical feedback and more visual feedback for storytelling and emotional self-expression (e.g. photos, drawings, stickers) may appeal to the “qualified self” rather than the “quantified self” that is commonly discussed in regard to PA self-monitoring [33, 34]. A novel way to use this self-monitoring as a form of motivation targeting integrated regulation among breast cancer survivors is narrative visualization (NV), a process of using annotation to help tell a story related to data presentation [35, 36]. NV uses drawings, photographs, and annotation to contextualize data. For example, one study used a scrapbook that included photographs and text annotating each photo and found that this process helped individuals find patterns in their daily arousal and relaxation [37]. Other investigators used an app that prompted users to regularly look at old photographs and add new annotations. These investigations found that this process led to greater reflection and improved well-being [38]. The annotation of photographs was also more acceptable and effective at clarifying personal values than written survey methods [39]. A growing cross-disciplinary evidence base suggests that adding visuals in the form of photographs and/or drawings can promote engagement with and reflection on data; thus, a PA intervention targeting breast cancer survivors that uses an NV strategy may be successful in improving long-term PA behavior.

We developed an intervention built upon self-monitoring techniques using wearable devices to include narrative visualization techniques that targeted integrated regulation for improving PA among breast cancer survivors. Because adherence is particularly low among older breast cancer survivors, we focused this pilot study on those over age 55 [11]. The purpose of this study was to explore the feasibility of using of scrapbooking activities as an NV strategy for older breast cancer survivors over a 7-day period. We also investigated themes from the completed scrapbook pages to explore the impacts of PA on their daily lives and to refine intervention materials for future studies.

## Methods

### Study design and population

Twenty participants were recruited based on the following eligibility criteria: [1] age between 55 and 79 years, [2] self-identify as female, [3] self-reported diagnosis of breast cancer, [4] no self-reported disabilities or other physical barriers to performing PA, [5] report < 150 minutes of moderate-vigorous physical activity per week, [6] able to read and understand English, [7] daily access to a smartphone or similar device compatible with the self-monitoring app used, and [8] cleared to participate as determined by the Physical Activity Readiness Questionnaire+. Sociodemographic information was self-reported to study coordinators during initial screening. The study protocol was approved by the university’s institutional review board, and all participants provided written informed consent.

### Procedures

**Fig. 1:**
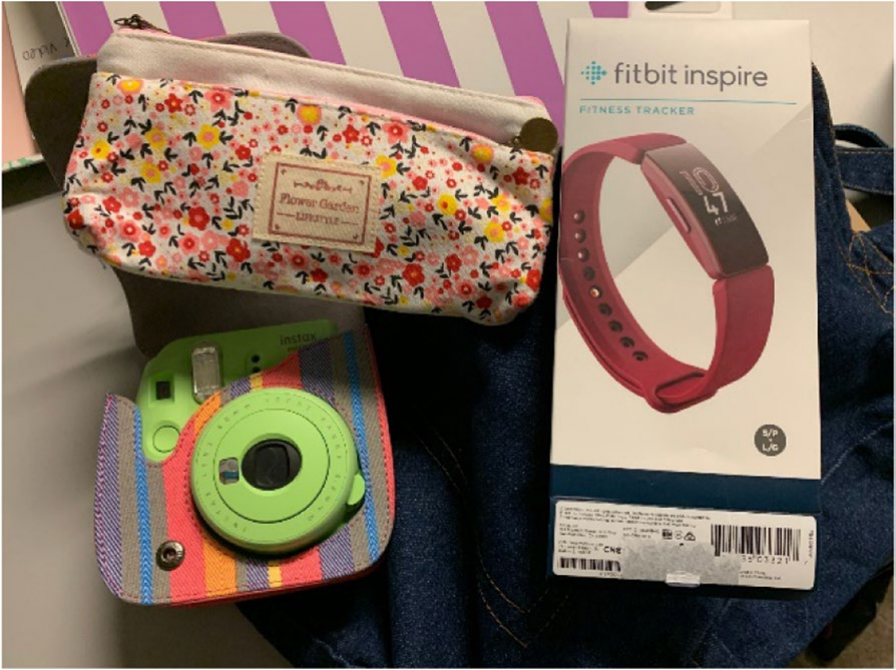
Instant camera, activity monitor, and art supplies. (Note: this photograph was taken by the investigators)

Participants were given an instant camera with film (FujiFilm Instax or Kodak Printomatic) to take photographs that reflect their values, a decorative carrying bag, and a workbook containing pages to attach photographs and document their daily PA over the 7-day period. To artfully decorate their photos and daily PA logs, participants were also provided various art supplies such as stickers, colored pens, photo corner stickers and decorative adhesive tape (Fig. 1). Stickers included sheets of various emojis (e.g., smiley faces, food, hearts), positive affirmations (e.g., “You go girl!”, “Believe In Yourself”), and fitness-themed affirmations (e.g., running shoes, bicycles, dumbbells). The fitness and affirmation stickers came from standard packs of planner stickers, such as those available from The Happy Planner (https://thehappyplanner.com/) and Avery (https://www.avery.com/) companies.

Additionally, participants received a wearable activity monitor (Garmin Vivoactive or FitBit Aspire) to monitor their daily steps. The purpose of the activity monitor was not to objectively assess their step count but subjectively measure participants’ experience with using the device. Participants were assisted in fitting the monitor onto their wrists and installing the associated software (either Garmin Connect or Fitbit) onto their smartphone. Participants were also shown how to access their step data so they could log their daily activity into their workbooks. The paper workbook consisted of two parts: an introductory values clarification exercise and daily logs. The initial values clarification exercise asked participants to take 10 photographs using the instant camera reflecting their matters of importance in their everyday life [39]. They were purposefully not provided detailed instructions to allow for their own interpretation. They were then asked to affix the photographs into their workbooks using the provided supplies and asked to write out notes or annotations about what those photographs represented and how they reflect things they value in life. For the daily logs, a blank graph was provided so participants could draw their daily progress in terms of steps taken over the course of the day along with any annotations or stickers they wanted to use. In addition, a set of three questions each day was provided for participants to assess how active they were, why they think they were or were not active, and to elaborate on what impacted decisions about activity. Since the focus was on the feasibility of using of the materials provided, participants were not given any recommendations for daily PA goals; instead, they were encouraged to write notes, indicate times activities started or ended, and use stickers to show what happened throughout the day, how they felt, or anything else they would like to record. Reflection questions were provided each day for participants to reflect on their activity at the end of the day, as well as after the 7 days as shown in Table 1.

**Table 1:** Reflection prompts

Timing	Prompt
Daily	How were you active today?
	Why do you think you were (or were not) active today?
	What impacted your decisions about activity today?
After 7 days	Looking back, do you see any patterns in your activity?
	How can you take this knowledge and use it to be more active next week?
	How do you feel about your progress this week? Do you feel closer to your goals?

Participants were given the choice to use or not use whatever materials they preferred over the 7 continuous days. Workbooks were then returned, and the pages subsequently digitized for analysis.

### Data analysis

We chose to treat workbook pages in a method similar to the products of photovoice interventions, which also include expressive photographs and text [40]. Content analysis of stickers was conducted by two independent coders, abstracting information on number of stickers and logging each sticker by an assigned name (e.g., heart eyes smiley, rolling eyes emoticon). The two coders met to assign emojis to the categories of positive (smiles for mouth, hearts for eyes, etc.) or ambivalent/negative (frowns or lines for mouths, etc.). A hybrid thematic analysis approach, including purely inductive analysis as well as deductive analysis based on a priori themes expected based on the theoretical framework of the intervention (SDT) that informed coders’ thinking was used to analyze photographs, drawings, and written content provided by the participants (NVivo 12 Pro, QSR International). We were testing intervention materials that were created specifically to target theoretical constructs; hence, we expected responses related to those constructs. Further, we anticipated responses based on previous responses in earlier pilot studies of similar materials that formed the basis of this intervention. This approach resulted in a priori themes from SDT related constructs which included: social relationships, mastery/growth, spirituality. Because this qualitative analysis was performed specifically as part of a process of intervention materials refinement, it was necessary to include a deductive element. This approach was pragmatic rather than post-positivist or constructivist, with a focus on discovering information that would be effective for refining intervention materials for future studies. Codes were developed by two independent coders (JRB and EJL) by identifying people, places and things that were photographed, and by iteratively reading through the comments made by participants to identify recurring themes. The inductive codes were developed based on specific values and identities mentioned by the participants; hence, all codes were related to the SDT concept of integrated regulation. To ensure methodological rigor, a the coders met regularly to negotiate the codebook during the iterative coding process, and a final meeting was held to resolve all differences in coding. Then, illustrative quotes were selected for each major theme identified. The intervention was deemed feasible if the participants successfully completed the procedures as instructed. Again to ensure methodological rigor, the qualitative and quantitative portions of this analysis were performed sequentially, with the quantitative investigation of the stickers coming first, followed by qualitative investigations of photographs followed by the text. In addition, synthesis was sequential, with the quantitative content analyses occurring first because of the nature of the questions. The quantitative feasibility questions (will participants use these materials) needed to be ‘yes’, essentially, for the qualitative analyses (‘how do they use the materials’) to be possible. Findings regarding sticker use informed the coders’ thinking regarding themes when reviewing the text of the workbooks. Finally, quantitative analyses were not used to interpret themes developed from the qualitative portion as these methodologies represent different aspects of the data collected; however, both were used in the overall synthesis of findings.

## Results

Of the 20 consented women (mean age 67 ± 5 years, 45% non-Hispanic white), 1 participant was lost to follow-up due to medical complications, 1 did not return phone calls, and 1 was unable to complete the procedures due to cultural barriers (preferred to communicate via spouse). Since the latter participant chose to let her male spouse answer the questions regarding feasibility and preferences, we judged her data not to be interpretable as personal reflection and the intervention not culturally appropriate for her.

### Quantitative results

**Fig. 2:**
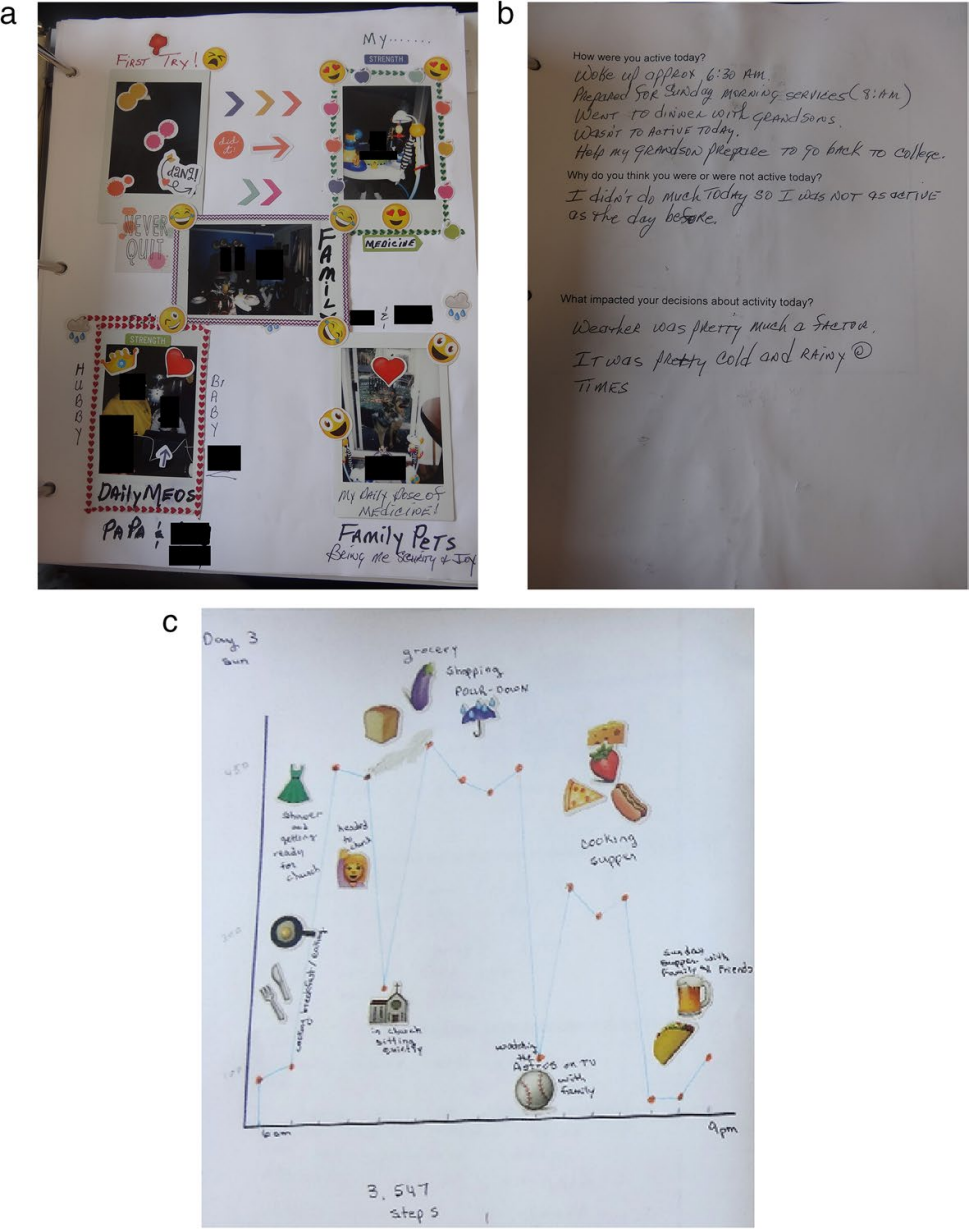
Example workbook pages with faces obscured

The NV procedures using both photos and written prose were universally accepted. Participants took a mean of 9 photos over 7 days (range: 4–10) and completed workbook questions regarding current PA and PA goals (Fig. 2). All participants completed all of the daily pages, though one printed her own pages that combined the two (so she had 8 pages instead of 15) and one forgot to do the weekly log at the end. Though all the daily pages were completed, not all were completed in the way we expected. Three participants drew bar charts rather than line charts, 1 printed out the line chart from the app and pasted it in the workbook, one wrote in numbers on the chart with no line, one made a bar chart on her computer then printed it and pasted it in, and one pasted photographs instead of a chart.

**Fig. 3:**
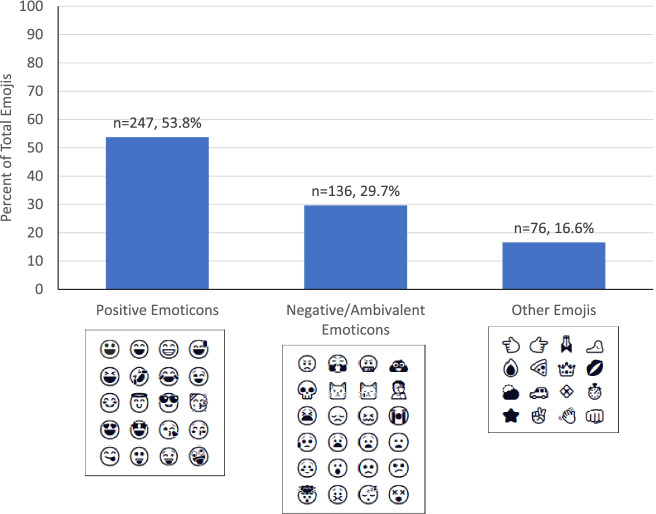
Emoji sticker usage

There were a total of 945 stickers used by all participants (mean ± SD per participant: 56 ± 49; range: 0–180). Almost half of these were emoji stickers (48.6%), most of which were positive themed (e.g., smileys, winks, laughing, blowing kiss, halo, star-struck, face with tongue, zany face, open hands, etc.); of the 459 emojis, 136 (29.7%) were negative themed (e.g., frown, anguished, worried, tired, disappointed, crying, etc.) (Fig. 3). There were also a substantial amount of stickers used that reflected positive or exercise-related sayings (*n* = 228, 24.1% of the total stickers; e.g., “Live Healthy”, “Just Do It”, “Be Strong”).

### Qualitative results

#### Photos

All participants successfully took photographs using the instant cameras and pasted at least one photograph into the workbook. Several participants included their failed photographs in addition to later, more successful tries. The most commonly occurring themes in the photographs were family, specific locations, everyday objects that held personal significance, religion, and friends.

Photos of family included spouses, children and grandchildren, other immediate family members, “chosen” family (i.e., individuals unofficially adopted into the family), and pets. A participant wrote next to photographs of her family, “My babies, my loves and who give my life meaning they make me laugh – they have been with me through my cancer fight.” Another participant wrote of her pet dog, “She loves me unconditionally, and reminds me of how much I love animals and how awesome I felt when I could save her. She is saving me now – she doesn’t leave my side. It’s important that while my world is crashing, I remember hers revolves around me, so I have to get my stuff together!”

Specific locations included photos of participants’ home, churches, gardens, the beach, and interesting places they passed. One participant pasted a picture of a tree on her walking path and wrote, “It is old with many branches like me. Older – many branches of my life. Peaceful and quiet. I feel good looking at it – peaceful – I love the shade as if God is covering me.” Another participant wrote, “This is the fountain in front of [name redacted] church. I brought my mom to church everyday, and we loved this fountain. It is peaceful to just sit by it. Mom passed away [date redacted] without ever knowing I have cancer. I sat here today after taking this picture & ‘talked’ to mom about it.”

Everyday objects included beds, books, clothing, flowers, computers, hats for local sports teams, etc. A participant wrote, “These roses are very meaningful to me. I planted them when I was sick on treatment. They blossomed all winter and have given the new hope ‘I can blossom in & out of season.’”

Religious photos often overlapped with other themes, such as specific locations and everyday objects (e.g., churches and Bibles). One participant took a picture of her church foyer and wrote, “I took this while attending church. My faith i [s] very important to me and a big part of my life, beliefs, values, morals, and salvation!” Another used an image of the inside of her church and wrote, “Religion. My church. Such an important part of my life. Growing in faith connecting with the lost, and helping those in need as we communicate God’s word through education, sacraments, worship, praise, and fellowship.”

While friends were often mentioned along with family, there were some photos where friendships were highlighted. For example, one participant wrote, “Friendship. I look at this picture and see trust, a mentor, a person so different from me, but so like me. Everyone needs a [name redacted]!” Another participant captioned, “One of my oldest friends. I’ve known him since he was 6 years old. Son of my best friend. Love this man! Love remembering the good times we had with him and his wife (and mother and dad when they were alive).”

#### Written text in the “values clarification” section and “daily/weekly activity” pages

To connect values from the photographs to daily PA, thematic analysis of written text in the values clarification section and text and drawings from the daily reflections revealed that participants felt that family had the largest impact on their PA. This was followed by: chores and obligations, health and illness, personal reflection, hobbies or activities, and shopping. These themes were discussed in the context of important aspects of the participants’ lives and also in journal form as things that occurred during the course of their days. For example, a participant might discuss religion as important to her and paste a photo of her Bible in the values clarification section, then mention going to church in her Wednesday and Sunday journal entries. Illustrative quotes of these concepts are provided in Table 2.

**Table 2:** Major themes found in participants’ written workbook text

Theme	Illustrative quotations
Family	“I walked a bit, stretched a little and also played kickball with family first time ever! Excited to be so active & feel great afterwards.”
	“I would love to see all of my grandkids grow up. I keep that as my motivation. Grandbaby on the way and I have GOT to be able to get to know this bundle.”
	“My health and family are primary. Stay busy & active will keep me with them and remind of what I’m living for.”
Chores and Obligations	“I woke up and fixed breakfast and did the household chores. Sit for a while and then went out with my daughter.”
	“Weekend household chores. Yard work … again.”
	“… ran a few errands, attended a meeting – walked from the garage back and forth.”
Health and illness	“Not very active. Had chemo all day.”
	“Back pain. It’s difficult to know how much walking to do without causing pain elsewhere. Drinking a lot of water forced me to get up and move to go to the bathroom.”
	“Not much activity. I did not feel good on this day. I started taking a new med. Doctor said medicine would make me feel sluggish until it adjust to my body.”
Personal reflection	“Looking through my pictures made me realize how blessed I am to have these wonderful gifts in my life. The actual visualization of these blessings, all together in this notebook make me understand the importance of them in my life and recognize the fact that I don’t tell them (enough) how important they are to me. God and family are what’s more important to me (fur babies included).”
	“‘Live like you were dying’ – we all have an expiration date, hope with more activity mine is not too soon.”
	“This study has allowed me a way to express my day – my feelings my thoughts. I needed this to show me the way to include a new activity in my life. I need to write and sort my activities and emotions. Help me to discover the me at age 66, a senior elderly. With old memories and making new memories. Building a new chapter. Learning how to live with the fear of cancer, recovering or being a survivor. Taking a closer look at my world – looking a [t] grass, sky, family, friend in a deeper way. Then attempt to put it all down to try and find out who I want to be when I grow up!”
Hobbies or Activities	“The activities of interest are most important – flowers that became a hobby when I needed something, ministry which is at my core and shopping.”
	“My ministry activities are very motivating because if I’m not healthy I cannot serve in ministry.”
	“Family project … Garden. I loved watching my husband teach my grandson how to use tools when they built this garden. So many lessons you can teach children when they don’t even have a clue that it’s a teaching session.”
Shopping	“I still had one more promise to keep. I went to the grocery store to get things for the meal for the Cowboys-Saints game party at my sister’s house. Big pot of chili and all of the fixins! WHEW!”
	“Today I needed a ‘me’ day to shop off the island and also to take care of some business however I was determined to get my steps in by pounding the pavement.”
	“My daughter loves to shop, of course we went to the mall. I walked for a while, going in and out of stores, we started at 3:00 PM and did not get home until 9:00, you know it was busy.”

Regarding the family theme, grandchildren were repeatedly mentioned in the workbooks as being significant factors for PA. There was also an emphasis among several participants on individuals who were chosen to be family by the participants. Several participants discussed friends who were instrumental in their health goals, and they often discussed the friends in terms of their importance regarding their commitments or obligations.

Hobbies and activities (not directly related to PA) were also frequently mentioned as part of their personal reflection. Hobbies and activities included gardening, shopping, and service to community via ministry or volunteering.

## Discussion

The purpose of this study was to explore the feasibility of scrapbooking activities as an NV strategy for older breast cancer survivors and identify themes from the completed scrapbook pages to explore the impacts of PA on their daily lives. This formative research revealed that the NV procedures, which are part of an intervention to increase integrated motivation regarding PA, were successfully utilized by all the breast cancer survivors in this study. These survivors understood the workbook instructions and were able to use the provided supplies as intended. Participants took approximately 9 of the expected 10 photos for the week—again, showing evidence for feasibility. Moreover, they also completed all of the expected 15 workbook pages, except for one missed weekly log page, and used an unexpectedly large number of stickers to illustrate their pages.

Regarding the utilization of supplies provided, considering that participants were instructed to record and annotate their daily PA in their workbooks, the high frequency use of fitness and motivation stickers was not surprising. However, it was surprising that they chose to use near equal numbers of positively and negatively themed emoji stickers possibly due to widespread use of emojis in modern text communications and a related feeling of comfort using them to express feelings, both good and bad [41]. In addition, cancer survivors tend to experience a large range of emotions as they adjust to life after cancer, particularly given the painful treatment process and related negative side effects [42]. Perhaps capitalizing on their positive emotions and giving meaning to their negative emotions could increase autonomous motivation to improve PA by building personal resources for coping (ref); thus, using NV may assist participants in contextualizing their feelings and their activity levels. In fact, enhancing positive emotions has been shown to increase adherence to PA (ref). With this in mind, the tendency for the breast cancer survivor population to use both negative and positive emojis to communicate their feelings warrants further study.

Several participants experienced a learning curve when using the instant cameras for the values clarification portion of the workbooks; since the cameras were preloaded with 10-packs of film when dispensed, there were usually fewer than 10 photos included in the workbooks. However, all participants expressed how they enjoyed and valued this part of the study. Huldtgren et al. suggested that decision making could be improved by focusing on personal values; similarly, the photographs taken as part of our study inspired the participants to reflect on their values [39]. In addition, Issacs et al. found their participants to benefit from photojournalism since the images served as reminders of positive experiences [38]. Here, photos of participants’ family were often featured, which agrees with the literature showing family to be a significant motivator for PA adoption and maintenance [33, 34]. These investigators also found friends to be significant motivators, again similar to our findings.

Participants used nearly all the space provided for them to draw their daily step charts and to answer questions about their daily activity. These results are in line with previous findings in the communications and human-computer interaction fields, which showed that individuals who self-monitored wanted options for storytelling and emotional self-expression [43, 44] and scaffolding to help them get from receiving new information to acting upon that information [45, 46]. Participants used photos, drawings, stickers, and text to reflect on their values as well as their identity, integrating them with their PA data to tell stories about their lives [47]. This process, as mentioned previously, reflects what has been called the “qualified self,” as opposed to the “quantified self” that is commonly discussed in regard to PA self-monitoring [48, 49]. Robertson et al. found that cancer survivors’ preferences differed from current standards used in application development for mobile devices in that they preferred value-based rather than numeric goals [24]. They also preferred PA data to be interpreted and contextualized. Here, numerical PA data was similarly one part of a larger set of data that described our participants’ attitudes towards PA and how it fit in with other mundane aspects of their day-to-day lives such as chores, shopping, and daily non-PA related activities. Further, they discussed these stories in the context of larger issues, such as family obligations, religion, and the impact of illness on their lives.

Weight loss was not identified as a major theme, though it is often the primary theme of many PA-related programs. Breast cancer survivors in this study were more focused on spending time with family and felt that improving their physical health could improve the quality of this time. They also focused on how their health impacted their PA. For example, several participants commented on how going to physician appointments or helping others go to their physician appointments negatively impacted their ability to do PA. In addition, whether they felt good or bad after these appointments also directly impacted their attitude toward their own PA goals.

These findings have several potential implications for future studies. PA interventions among the cancer survivor population should consider how activity monitors are utilized by the participants. While this study was not designed to document these effects, Lazar et al. suggest that numerical data, particularly those focused on calories or steps, may be less meaningful to most participants than visual data such as graphs and progress charts [50]. Activity monitor manufacturers provide several types of visual data in their applications, but current research rarely focuses on these aspects.

The tactile nature of our visualization procedures also suggests several potential areas of future research. The stickers we provided were extremely popular. It is not clear whether virtual stickers would have a similar impact to the tactile feeling of sticking a physical sticker to a piece of paper. We adopted a hybrid approach to this study, with electronic data collection but hard copies of all scrapbooking materials. As part of this decision process, we opted to use instant cameras with printed out photos rather than digital photos. In addition to comparing actual quantitative activity data, future studies may wish to investigate differences between purely digital scrapbooking, for example using an app like Day One (https://dayoneapp.com), as opposed to tactile scrapbooking such as was used here.

While the feasibility of this novel NV technique and insights gained from the utilization of the materials as noted above are the strengths of this study, it also had some limitations. First, this was a formative study focused on the basic feasibility of a novel intervention and thus, interpretations are limited by the small sample size. Second, since the primary goal was to simply evaluate utilization, the duration of usage was relatively short (7 days). Additionally, since this study was focused on the feasibility of using the materials, no measurement for motivation was obtained. Third, an unexpectedly large proportion of participants (three of the twenty) were lost to follow up despite this being a very short-term study. While this was unexpected and is unusual based on previous studies, we believe that the loss of contact with one participant and the loss of another to an acute health issue were likely chance occurrences. The third lost participant provided important information in that the scrapbooking activities were culturally inappropriate for her, which provided insight for future studies in this population (e.g., exclusion criteria added during recruitment, or adaptation of NV strategies to be more broadly culturally acceptable or tailored to specific cultures). Finally, since our target population is older women breast cancer survivors, generalizability is limited in age and gender. Additional research is needed to investigate whether these materials and procedures are feasible in other populations.

## Conclusions

In conclusion, the materials provided to the breast cancer survivors allowed them to successfully use NV techniques to reflect on their PA data and behavior. These techniques warrant further investigation as potential tools for promoting integrated regulation in activity monitoring interventions.

## Data Availability

De-identified data from this study are not available in a public archive; interested researchers may request access to the text portions of the data set per IRB standards by emailing the principal investigator (ellyons@utmb.edu).

## Abbreviations

BC: Breast Cancer
NV: Narrative Visualization
PA: Physical Activity
SDT: Self-Determination Theory
